# *Cordyceps bassiana* inhibits smooth muscle cell proliferation via the ERK1/2 MAPK signaling pathway

**DOI:** 10.1186/s11658-016-0023-z

**Published:** 2016-10-21

**Authors:** Enze Jin, Seongho Han, Mina Son, Sung-Whan Kim

**Affiliations:** 1 Department of Cardiology, The Fourth Affiliated Hospital of Harbin Medical University, Harbin, China; 2 Department of Family Medicine, College of Medicine, Dong-A University, Busan, Republic of Korea; 3 Department of Medicine, College of Medicine, Catholic Kwandong University, Gangneung, Republic of Korea; 4 International St. Mary’s Hospital, 25, Simgok-ro 100beon-gil, Seo-gu, Incheon, 404-190 Republic of Korea

**Keywords:** Cordyceps, ERK pathway, Inhibitory, MAPK, Mechanism, Natural product, Neointimal formation, Proliferation, Smooth muscle cells, Vascular

## Abstract

*Cordyceps* belongs to a genus of acormycete fungi and is known to exhibit various pharmacological effects. The aim of this study was to investigate the effect of *Cordyceps* species on the proliferation of vascular smooth muscle cells (VSMC) and their underlying molecular mechanism. A cell proliferation assay showed that *Cordyceps bassiana* ethanol extract (CBEE) significantly inhibited VSMC proliferation. In addition, neointimal formation was significantly reduced by treatment with CBEE in the carotid artery of balloon-injured rats. We also investigated the effects of CBEE on the extracellular signal-regulated kinase (ERK) signal pathway. Western blot analysis revealed increased ERK 1/2 phosphorylation in VSMCs treated with CBEE. Pretreatment with U0126 completely abrogated CBEE-induced ERK 1/2 phosphorylation. In conclusion, CBEE exhibited anti-proliferative properties that affected VSMCs through the ERK1/2 MAPK signaling pathway. Our data may elucidate the inhibitory mechanism of this natural product*.*

## Background

Atherosclerosis is a disease characterized by thickening of the inner portion of the artery walls [1]. One of the main pathological characteristics of this disease is the proliferation of vascular smooth muscle cells (VSMC). Recent studies have demonstrated a close relationship between restenosis and VSMC proliferation in the intimal layer due to accumulation of cells and extracellular matrix [2].

*Cordyceps* belongs to a genus of acormycete fungi that includes about 400 species [3]. Representative *Cordyceps* species are C. sinensis and C. militaris, which are have been used as traditional medicine for various diseases in Asian countries. These exhibit various pharmacological effects including anti-inflammatory, antioxidative, antidiabetic, and anticancer properties [4].

Cordycepin inhibits VSMC proliferation [5]. However, its anti-proliferative effects in VSMC and the underlying mechanisms of the *Cordyceps* species have yet to be addressed. We investigated the anti-proliferative activity of *Cordyceps* species extracts in VSMC and studied the underlying signal transduction mechanism. Therefore, our study investigates that the molecular mechanism underlying the anti-atherosclerotic effects of *Cordyceps* species extracts. This pathway might highlight a potential therapeutic intervention for cardiovascular disease.

## Methods

### Materials

*Cordyceps bassiana* ethanol extract (CBEE), *Cordyceps militaris* butanol extract (CMBE), and *Cordyceps pruinosa* methanol extract (CPME) were extracted using conventional extraction methods [6]. The signal pathway inhibitors U0126, SP600125, and SB202190 were purchased from Calbiochem (La Jolla, CA, USA). Antibodies against ERK, phospho-ERK, JNK, phospho-JNK, p38 MAPK, and phospho-p38 MAPK were purchased from Cell Signaling Technology (Beverly, MA, USA). Horseradish peroxidase (HRP)-conjugated goat anti-rabbit IgG was used as the secondary antibody.

### Solvent extraction

Solvent extraction was performed as described previously [6, 7]. In brief, Air-dried *Cordyceps* (175 g) were powdered and extracted at 70 ~ 80 °C with 80 % ethanol, methanol and butanol. The extract was filtered, taken to dryness under reduced pressure and concentrated by a rotary vacuum evaporator (EYELA N-1000, Japan). The lyophilized crude extracts were sequentially fractionated with *n*-hexane (Hexane), ethyl acetate (EtOAc) and *n*-butanol (BuOH). Each extract was dissolved in dimethyl sulphoxide (DMSO) as a concentration.

### Cell culture

Rat aortic smooth muscle cells (RaoSMCs) were cultured in DMEM supplemented with fetal bovine serum (Gibco, Grand Island, NY, USA) in 75 cm^2^ flasks in a 37 °C incubator at 5 % CO_2_.

### Cell proliferation assays

RaoSMCs were plated in triplicate in 96-well plates at 2 × 10^3^ cells per well. The cells were starved with 0.1 % FBS for 24 h and then treated with three kinds of *Cordyceps* extracts or 10 % serum. After treatment, cell proliferation was measured using a CCK-8 assay kit (Dojindo, Japan). The CCK-8 assay kit was treated with DMEM, and then 100 μl was added to each well and incubated for 2 h at 37 °C. The absorbance was measured at 450 nm with a spectrometer.

### Neointima formation assay

Animal experiment conforms to the Animal Care and NIH guidelines were approved by the local animal care committee. Balloon denudation of the common carotid artery endothelium was evoked in four-week old 250 g Male Sprague–Dawley rats (Daihan-Biolink Co., Chungbuk, Korea) as previously reported [5, 8]. All rats were anaesthetized with Ketamine (10 mg/kg, Yuhan, Seoul, Korea) and Xylazine (5 mg/kg, Bayer Korea, Seoul, Korea). After exposure of the left carotid artery, a 2F Fogarty balloon catheter (Edwards Lifesciences, Mississauga, ON) was inserted into the external carotid branch to the aortic arch. And then catheter was insufflated to produce slight resistance, and withdrawn three times. Sham-operated control was performed with the same procedure and the exception of the balloon insertion. Vehicle (saline) or 0.05 ml of CBEE (4 μg/ml) was administered daily via ip. for 3 weeks after the balloon injury.

### H&E staining

Sprague–Dawley rats were sacrificed 3 weeks after the balloon injury. And their carotid arteries were harvested and fixed with 4 % paraformaldehyde. Within 24 h after fixation, each section was embedded in OCT compound (Sakura Finetek USA, Torrance, CA, USA), snap frozen in liquid nitrogen, and sectioned in thickness increments of 10-μm carotid artery sections were cut with a microtome and mounted on slides. Sections were stained with hematoxylin and eosin (H&E) and evaluated with light microscopy to assess the histological effects. Normal and neointimal areas were measured using NIH images.

### Western blotting

The western blot analysis was performed as previously described [9, 10]. Cells were washed once with PBS and then protein was extracted using a lysis buffer (Biosesang, Korea) containing 150 mM NaCl, 1 % Triton X-100, 1 % sodium deoxycholate, 0.1 % SDS, 50 mM Tris/HCl (pH 7.5), and 2mM EDTA (pH8.0) with a protease inhibitor cocktail (GenDEPOT, Barker, TX, USA) and phosphatase inhibitor cocktail (Sigma, St. Louis, MO, USA). Protein concentrations were determined using a BCA protein assay kit (Pierce Biotechnology, Rockford, IL, USA). For western blotting, equal amounts of protein were electrophoresed on 12 % sodium dodecyl sulfate (SDS)–polyacrylamide gels, transferred to nitrocellulose membranes (BioRad, Hercules, CA, USA), blocked using TBS-T (TBS-Tween 20; 0.1 % Tween 20) containing 5 % (w/v) non-fat dried skim milk powder for 1 h at room temperature, and incubated with monoclonal primary antibodies (1:1000) in a blocking buffer overnight at 4 °C [8, 11]. The membrane was washed three times with TBS-T for 10min and incubated with horseradish peroxidase (HRP)-conjugated secondary antibody for 1 h at room temperature. After extensive washing, bands were detected using ECL western blotting detection reagent (Biosesang). Protein expression levels were determined using the Davinch-K western image System (Lab plus, Korea).

### Statistical analysis

All data are presented as mean ± standard deviation (SD) from at least three independent experiments. The statistical significance was determined using the Student’s *t*-test for unpaired samples between two groups. *p <* 0.05 was considered statistically significant.

## Results

### CBEE inhibited VSMC proliferation

**Fig. 1:**
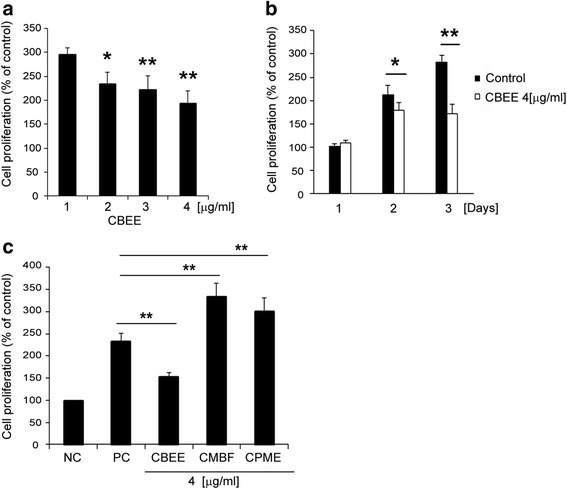
Figure 3. Effects *Cordycep* species on SMC proliferation. Effects of CBEE on SMC anti-proliferation (**a** and **b**). We evaluated dose and time course responses on SMC anti-proliferation of CBEE. **a** Dose dependent SMC anti-proliferation effects of CBEE. Cell counts were conducted after 3 days of culture. **b** Time dependent SMC anti-proliferation effects of CBEE. *p < 0.05. **p < 0.01. *n =* 3 per group. **c** Effects of three *Cordycep* species on SMC proliferation. Serum-starved SMC served as the negative control (NC) and SMCs treated with 10 % fetal bovine serum (FBS) were the positive control (PC). Data are presented as percent change in negative NC. Cell counts were conducted after 3 days of culture. **p < 0.01 vs PC. *n =* 3 per group

To confirm the effect of CBEE on VSMC anti-proliferation, cells were cultured for 24 h in complete medium containing 10 % FBS at various concentrations of CBEE (0 ~ 4 μg/ml). As shown in Fig. 1a, we observed a concentration-dependent decrease in cell viability after treatment with CBEE. In addition, CBEE exerted a time-dependent decrease in cell viability (Fig. 1b). We could not find any Toxicity of CBEE in this study.

### Anti-proliferative of *Cordyceps* species on VSMC

To identify the anti-proliferative effects of other *Cordyceps* species, VSMC were cultured for 3 days in complete medium containing 10 % FBS with three *Cordyceps* species: CBEE, CMBF, and CPME. A cell proliferation assay showed that CBEE significantly inhibited VSMC proliferation (Fig. 1c). However, other *Cordyceps* species did not inhibit VSMC proliferation.

### CBEE inhibited neointima formation

**Fig. 2:**
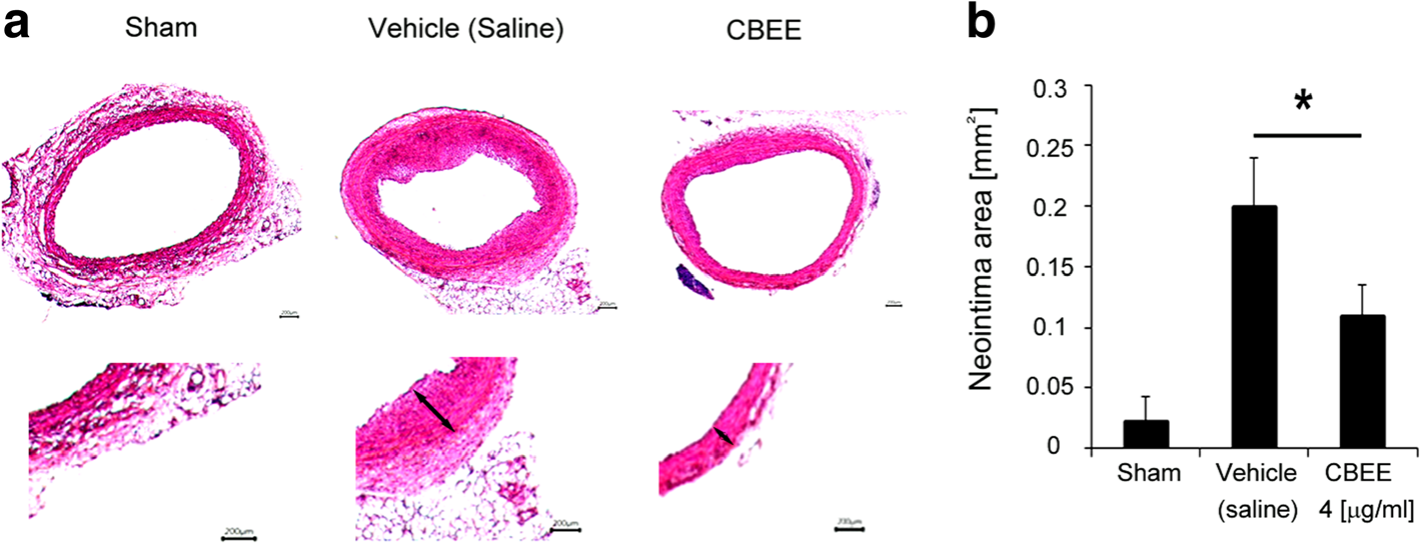
Effects of CBEE on neointimal formation. Saline or CBEE was treated for 3 weeks after balloon injury. Representative photographs of H&E staining (**a**) and neointima quantification were shown in bar graph (**b**). *Arrows* indicate neointima. *p < 0.05. *n =* 4 per group

To quantify the effect of CBEE on neointimal hyperplasia, we induced balloon injury in rats. Histomorphometric analysis using carotid arterry sections was performed. Vehicle treated group induced abundant neointimal hyperplasia. However, CBEE (4 μg/mL) treated group exhibited a significant reduction of neointima formation compare with control (vehicle) group (*p =* 0.019) (Fig. 2).

### U0126 inhibit CBEE-induced VSMC anti-proliferation

**Fig. 3:**
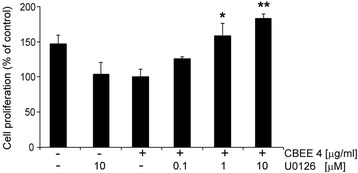
Effects of U0126, a specific ERK1/2 inhibitor, on CBEE-induced SMC proliferation. SMC were treated with the MEK/ERK inhibitor U0126 for 1 h and were then stimulated with CBEE for 72 h. Proliferation assays were performed with the CCK-8 assay. *p < 0.05. **p < 0.01 vs CBEE-treated cells. *n =* 3 per group

To better understand the molecular mechanisms involved in the VSMC anti-proliferative effects of CBEE, we investigated the possible involvement of the MAPK signaling pathway. To determine whether extracellular signal–regulated kinase (ERK) 1/2 activation is involved in CBEE-induced VSMC proliferation, we treated cells with the MEK/ERK inhibitor U0126 for 1h and then stimulated with CBEE for 72 h. Proliferation assays were then performed with the CCK-8 assay. The ERK1/2 inhibitor U0126 significantly blocked CBEE-mediated VSMC anti-proliferation in a dose-dependent manner, as examined by cell counting (Fig. 3).

### CBEE-activated ERK1/2 phosphorylation

**Fig. 4:**
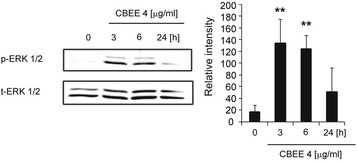
Time course response of ERK activation in SMC treated with CBEE. A western blot was analyzed for phosphorylated ERK1/2 (p-ERK) and total ERK1/2 (t-ERK1/2). **p < 0.01 vs untreated SMC. *n =* 3 per group

Our previous results prompted us to further investigate whether CBEE activates the ERK 1/2 signaling pathway. To address this issue, cell lysates were probed for ERK 1/2 phosphorylation and protein levels. Western blot analysis revealed increased ERK 1/2 phosphorylation in VSMCs treated with 4 μg/mL CBEE after 3, 6, and 24 h, with the maximal increase occurring after 3 h of treatment (Fig. 4).

### CBEE-activated ERK1/2 phosphorylation is inhibited by U0126

**Fig. 5:**
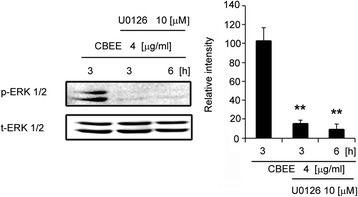
Time course response of ERK activation in SMC treated with CBEE and inhibited with U0126. A western blot was analyzed for p-ERK and t-ERK1/2. **p < 0.01 vs CBEE only treatment. *n =* 3 per group

Next, we examined the action of the specific inhibitor U0126 on the ERK 1/2 pathway activation. Western blot analysis showed that pretreatment with U0126 completely abrogated CBEE-induced (3- and 6 h) ERK 1/2 phosphorylation (Fig. 5).

## Discussion

Our results clearly demonstrate that CBEE inhibits VSMC proliferation. Notably, CBEE inhibited VSMC proliferation by modulating the ERK1/2 MAPK signaling pathway. To the best of our knowledge, this is the first report connecting molecular mechanisms of CBEE to its anti-proliferative effects on VSMC.

Endothelial injury initiates the pathogenesis of various vascular diseases such as atherosclerosis [12]. Chronic damage to arterial endothelium leads to lipid accumulation and adhesion of monocytes and platelets. Various cytokines derived from these cells promote the migration and proliferation of VSMC, leading to the later atherosclerotic plaque [1, 13]. Another form of occluded arteries in revascularization is restenosis. Although multiple therapies such as anticoagulants and antiplatelet agents have been developed, restenosis is still a challenging risk factor after percutaneous transluminal coronary angioplasty (PTCA) [14]. Thrombosis by elastic recoil and vasospasm is the major cause of early restenosis [15]. However, fibrocellular proliferation, which includes VSMC and inflammatory cells, is the main pathogenic cause of late restenosis [16]. Thus, we explored natural products as an anti-proliferative drug to treat vascular disease related to VSMC hyperplasia.

Recent reports indicate that cordycepin inhibits VSMC proliferation [5] and attenuates neointimal formation by inhibiting reactive oxygen species-mediated responses [17]. However, the anti-proliferative effects and biological activity of various *Cordyceps* species on VSMC are not fully understood. We found CBEE, which contained a yellowish colored compound, was reported to have significant anti-proliferative effects on VSMC. However, data on the biological activities of CBEE are limited. One recent report demonstrated that CBEE suppressed the expression of interleukin-12 and phosphorylation of p38 in lipopolysaccharide-activated macrophages without altering cell viability [18]. Interestingly, only CBEE showed inhibitory effect on VSMC in this study. We speculate that other treatment time or concentration of *Cordyceps* species extracts (CMBF or CPME) could exhibit inhibitory effect on VSMC. However, major active compounds derived from CBEE and their anti-proliferative or anti-inflammatory properties compare with CMBF or CPME should be clarified in further studies.

According previous reports, ERK1/2 has distinct functions in the inhibition of cell growth [19]. Jung et al. reported that the Ras/ERK1 pathway regulates G1-phase cell-cycle arrest in cordycepin-induced inhibition of VSMC proliferation [20]. Cordycepin also inhibited DNA synthesis and cell growth. In addition, cordycepin is associated with proliferative effects via the P38 and JNK MAPK signaling pathway in several cell lines [21, 22]. Consistent with this report, CBEE activated phospho-ERK and the ERK inhibitor U0126 inhibited CBEE induced anti-proliferative effects on VSMC, indicating a significant association with the ERK1/2 MAPK signaling pathway.

## Conclusion

In conclusion, CBEE exhibited anti-proliferative properties that affected VSMC through the ERK1/2 MAPK signaling pathway. Our data may elucidate the inhibitory mechanism of this natural product, *Cordyceps bassiana,* and helps us to understand its protective effects in cardiovascular disease-associated pathophysiology. However, further studies are necessary to identify the major active components of CBEE.

## Abbreviations

CBEE: *Cordyceps bassiana* ethanol extract
CMBE: *Cordyceps militaris* butanol extract
CPME: *Cordyceps pruinosa* methanol extract
ERK: Extracellular signal-regulated kinase
MAPK: Mitogen-activated protein kinase
VSMC: Vascular smooth muscle cells
